# Exploring the Role of Pituitary Adenylate Cyclase-Activating Polypeptide (PACAP) and Kynurenine Pathway Dysregulation in Migraine Pathophysiology Among Women With Polycystic Ovary Syndrome (PCOS)

**DOI:** 10.7759/cureus.71199

**Published:** 2024-10-10

**Authors:** Ozge Longwill

**Affiliations:** 1 General Practice, Private Practice, San Diego, USA

**Keywords:** cgrp, kynurenine, metabolism of tryptophan, migraine disorder, migraines and headaches, pacap, polycystic ovary syndrome (pcos), women's reproductive health

## Abstract

A narrative review was undertaken to explore the current understanding of the relationship between polycystic ovary syndrome and migraine headaches, with a focus on the potential roles of pituitary adenylate cyclase-activating polypeptide and the kynurenine pathway in the shared pathophysiology of these conditions. Emerging evidence suggests that pituitary adenylate cyclase-activating polypeptide may be a key player in the development of migraine headaches, with potential implications for the higher incidence of migraine observed in women with polycystic ovary syndrome. Additionally, dysregulation of the kynurenine pathway and altered levels of kynurenine metabolites have been linked to both migraine and polycystic ovary syndrome, indicating a complex interplay between hormonal, metabolic, and neurological factors in the comorbid presentation of these disorders. Further research is needed to elucidate the specific mechanisms underlying these associations and to develop targeted therapeutic approaches for managing migraine in the context of polycystic ovary syndrome.

## Introduction and background

Polycystic ovary syndrome (PCOS) is a common endocrine disorder affecting 6%-20% of women of reproductive age, characterized by hormonal imbalances, polycystic ovaries, and a range of clinical features. According to the Rotterdam criteria, the diagnosis of PCOS requires the presence of at least two of the following three features: oligomenorrhea or amenorrhea, clinical or biochemical signs of hyperandrogenism, and polycystic ovaries on ultrasound. Additionally, hyperprolactinemia is often observed in women with PCOS and can complicate the diagnosis and management of the disorder [1]. Emerging evidence highlights the dysregulation of the kynurenine pathway, a major route of tryptophan metabolism, as a critical factor in PCOS pathophysiology. Tryptophan, predominantly metabolized into kynurenine, plays a role in inflammation, metabolic dysfunction, and neuroendocrine regulation [2]. Migraine is a neurovascular disorder characterized by unilateral, throbbing or pounding headaches accompanied by photophobia, phonophobia, nausea, vomiting, and disability, typically lasting from four to 72 hours. In women, fluctuations in estrogen levels, particularly elevated estrogen, play a significant role in migraine pathophysiology. High estrogen levels can influence neurovascular mechanisms and promote inflammation, leading to increased susceptibility to migraines, especially during menstrual cycles and hormonal changes associated with conditions like PCOS [3], with or without aura which disproportionately affects women, with a prevalence three times higher than in men [4]. The trigeminal nerve plays a pivotal role in migraine pathophysiology, with key vasodilatory neuropeptides such as calcitonin gene-related peptide (CGRP) and pituitary adenylate cyclase-activating polypeptide (PACAP) contributing to the onset of migraine through neurogenic inflammation and vascular dilation [5,6]. PACAP is a versatile neuropeptide that belongs to the glucagon-growth hormone-releasing factor-secretin superfamily. It exists in two active forms: PACAP-27 and PACAP-38, with PACAP-38 being the predominant variant and highly conserved across species. This neuropeptide is known to influence learning, memory, and behavior by modulating neuronal growth, development, and repair. Additionally, PACAP has significant anatomical similarities with CGRP in brain regions linked to migraine, indicating that it may have a role similar to CGRP in migraine mechanisms. A cohort study in Denmark which includes over 19K women with PCOS found a higher prevalence of migraine in that patient population [7]. Another study investigated the comorbidities, medication use, and healthcare services among women with PCOS and reported higher rates of migraine in women with PCOS compared to the healthy population [8].

In recent years, emerging research has explored the interconnected roles of metabolic pathways, including tryptophan metabolism, in both PCOS and migraine. Tryptophan serves as a precursor to serotonin, a neurotransmitter targeted by migraine treatments, and the kynurenine pathway, which generates neuroactive compounds implicated in migraine pathophysiology [9]. Many kynurenines are neuroactive, modulating neuroplasticity and/or exerting neurotoxic effects in part through their effects on NMDA receptor signaling and glutamatergic neurotransmission [10]. A cross-sectional study identified that women with PCOS exhibited significantly elevated kynurenine levels, particularly in correlation with insulin resistance, obesity, and elevated inflammatory cytokines. This suggests that kynurenine pathway dysregulation may exacerbate PCOS metabolic disturbances and potentially contribute to associated comorbidities, such as diabetes and cardiovascular disease. Additionally, the activation of indoleamine 2,3-dioxygenase (IDO), a key enzyme in the kynurenine pathway, was linked to systemic inflammation in PCOS patients. The study highlights the need to explore kynurenine pathway metabolites as potential biomarkers for PCOS risk prediction and therapeutic targets [2]. The kynurenine pathway is regulated by, and in turn regulates multiple other physiological systems that are commonly disrupted in psychiatric disorders, including endocrine, metabolic, and hormonal systems [10]. Abnormal tryptophan catabolism has also been linked to PCOS, suggesting a possible common pathway between these two conditions [2].

PACAP is a neuropeptide involved in regulating reproductive functions, is elevated during migraine attacks and has been associated with reproductive dysfunctions in PCOS [11,12]. Studies have shown that women with PCOS are more likely to experience migraines, particularly during menstruation, highlighting the influence of hormonal fluctuations on this comorbidity [13].

There may be numerous potential mechanisms underlying the comorbidity between PCOS and migraine, including hormonal factors, metabolic disturbances, and neuroinflammation. This review aims to investigate the shared pathophysiological mechanisms of PACAP dysregulation and disruptions in the kynurenine pathway, exploring how these may contribute to the co-occurrence of migraine and PCOS in women. By examining these overlapping pathways, we seek to provide insights that could lead to more effective, targeted interventions for women suffering from both conditions.

## Review

Methodology

Research Design

The primary research question for this narrative review is:

“What evidence exists on the connection between pituitary adenylate cyclase-activating polypeptide and the kynurenine pathway about migraine prevalence among women with polycystic ovary syndrome?”

To address this overarching question, the review will delve into the following sub-topics:

1. The role of PACAP in the pathogenesis of migraine and its potential involvement in PCOS

2. The dysregulation of the kynurenine pathway and its relationship to both PCOS and migraine

3. The potential shared mechanisms underlying the comorbidity of migraine and PCOS

4. Implications for future research and clinical applications

Literature Review

The literature search was conducted using various electronic databases, including PubMed, Embase, and Cochrane Library, to identify relevant studies published in English. The key search terms included “polycystic ovary syndrome,” “migraine,” “PACAP,” and “kynurenine pathway,” and their combinations. Additionally, the reference lists of identified studies and relevant reviews were examined to determine articles that met the inclusion criteria.

After initial screening, 60 articles were selected for in-depth review based on their relevance to the research question and the quality of the study design. Review articles, animal studies, cross-sectional studies, meta-analyses, and original research articles were considered.

Inclusion criteria: Studies published from 1995 to 2024, studies published in English, review articles, animal studies, cross-sectional studies, meta-analyses, and original research articles were considered. Studies included were those that examined the potential links between PCOS and migraine, with a focus on the roles of PACAP and the kynurenine pathway.

Exclusion criteria: Studies not published in English and studies on children and abdominal migraine.

Data Extraction

Data are extracted independently from each study included. Sixty articles were selected for in-depth review based on their relevance to the research question and the quality of the study design.

Primary outcomes: Following are the primary outcomes:

1. Identify and summarize studies that explore the relationship between PACAP pathway disruption and migraine occurrence or severity in women with PCOS.

2. Compile evidence from existing literature on the alterations in the kynurenine pathway in women with PCOS and their potential role in migraine pathophysiology.

3. Analyze published data to evaluate the prevalence of migraine in women diagnosed with PCOS.

Secondary outcomes: Following are the secondary outcomes:

1. Review studies that discuss the biological mechanisms linking PACAP dysregulation and kynurenine pathway alterations to migraine pathophysiology in women with PCOS.

2. Examine literature discussing the impact of hormonal fluctuations typical in PCOS on PACAP and kisspeptin production and how these may affect migraine susceptibility.

3. Examine literature discussing the impact of dysregulation of the tryptophan/kynurenine pathway on migraines and/or PCOS.

Data Analysis

Extracted data are scientifically presented in the form of a narrative literature review. This detailed extraction aimed to offer a comprehensive understanding of the role of PACAP and kynurenine pathway dysregulation in migraine pathophysiology among women with PCOS.

PACAP: exploring its link to migraine and PCOS

PACAP plays a multifaceted role in female reproduction, particularly through its impact on follicle survival, steroidogenesis, and granulosa cell function. PACAP has been shown to promote follicle survival by suppressing apoptosis in rat ovarian tissue [14]. By promoting cAMP production and inhibiting apoptosis in ovarian tissue, PACAP contributes to follicle maintenance and oocyte maturation, mirroring the effects of luteinizing hormone [14,15]. In the context of PCOS, where granulosa cells exhibit reduced apoptosis and heightened responsiveness to follicle-stimulating hormone, PACAP's regulatory influence on these cells may be a critical factor in the pathophysiology of the condition [16,17]. Furthermore, PACAP has been shown to regulate the meiotic maturation of oocytes, mimicking the action of luteinizing hormone [18]. These insights suggest that PACAP could be a key modulator in the development of PCOS and offer potential avenues for therapeutic exploration.

PACAP has also been linked to migraine pathophysiology due to its role in neurovascular inflammation and vasodilation, both of which are significant triggers for migraine. In women with PCOS, hormonal imbalances-such as elevated androgens and insulin resistance-can exacerbate PACAP dysregulation, potentially intensifying migraine severity [19]. PACAP influences the trigeminovascular system, promoting CGRP release, which is known to provoke migraine attacks. Studies have shown that PACAP is elevated during migraine attacks in women, particularly in those with metabolic disorders like PCOS. For instance, recent research has demonstrated a correlation between higher PACAP levels and increased migraine frequency and intensity in PCOS patients, highlighting the involvement of hormonal and neuroinflammatory pathways in this population. This dysregulation not only contributes to more frequent and severe migraines but may also worsen PCOS symptoms, creating a cycle of pain and hormonal disruption [20].

Sex hormones may modulate CGRP release in the trigeminovascular system. A study found that women with PCOS exhibited higher plasma levels of CGRP compared to control subjects and these elevated CGRP concentrations were positively correlated with insulin and gonadal hormone levels in these PCOS patients [21]. Similar to CGRP, PACAP is a potent vasodilator and can induce migraine-like headache attacks when administered systemically [22]. What we know is that while CGRP and PACAP share similar functions, the PACAP pathway appears to be independent of the CGRP pathway, suggesting that they act in parallel ways to cause a migraine-like symptom [23].

Migraine attacks are also associated with elevated PACAP levels in plasma. Tuka et al.'s study on two rat models showed that PACAP-38-LI was elevated in the systemic circulation following electrical trigeminal ganglion activation [24]. This observation is consistent with the hypothesis that PACAP may be involved in the pathogenesis of pain disorders linked to trigeminal nerve activation. Later human studies by Tuka et al. provided the ﬁrst evidence that PACAP-38 concentration in the plasma of migraineurs is significantly lower in the interictal period as compared with that of healthy volunteers, but increases during migraine attacks relative to the attack-free period; the diﬀerence in the plasma levels of PACAP-38 in migraineurs in the two periods indicate that this peptide is involved in the development of the attacks [25]. Another study found that the intravenous administration of PACAP-38 induced headaches in healthy participants and provoked migraine-like attacks in migraine patients [26]. A study found that PACAP-38-induced migraine was associated with vasodilatation of the middle meningeal artery, and sumatriptan, which is a serotonin receptor agonist, was able to relieve the migraine pain by mirroring the contraction of the middle meningeal artery [27]. In a phase 2 clinical trial, a single intravenous administration of a monoclonal antibody targeting PACAP demonstrated superior efficacy compared to placebo in decreasing migraine attack frequency over the subsequent four-week period [28]. Results from this study suggest that further investigation of PACAP-targeted therapies for migraines is warranted [29].

Hypothalamic PACAP acts as a local regulator of GnRH release. The effect on GnRH may be through Kisspeptin neurons. Kisspeptin neurons play a prominent role in transmitting the regulatory signals that govern the pulsatile or surge-like release pattern of gonadotropin-releasing hormone [30]. Emerging evidence suggests that PACAP neurons modulate the kisspeptin-GnRH neuronal network by regulating the activity of Kiss1 neurons, which subsequently impacts fertility [31]. The research by Ross et al. indicates that mice lacking PACAP in the ventral premammillary nucleus exhibited fewer and longer estrous cycles, reduced fertility, and delayed pregnancy. However, these mice responded normally to kisspeptin, suggesting PACAP modulates kisspeptin release. Kisspeptin is a potent stimulator of GnRH neurons [32]. Research has found that women with PCOS have higher circulating levels of kisspeptin compared to healthy individuals [33] In women with PCOS, there is a positive correlation between circulating kisspeptin levels in the blood and the high levels of luteinizing hormone [34]. This observation may be attributed to heightened PACAP levels in women with PCOS, which may also contribute to the increased incidence of migraine in this population. PACAP may be involved in the differential release of luteinizing hormone and follicle-stimulating hormone observed in various physiological and pathological conditions, including PCOS, and certain pituitary tumors where high-affinity PACAP-binding sites have been identified [35].

Coutinho et al. found that acutely inhibiting kisspeptin neurons in a mouse model of PCOS effectively reduced the abnormally elevated LH pulsatile secretion [36]. Furthermore, chronically suppressing kisspeptin neuron activity reversed the previously high LH and testosterone levels, restoring them to healthy control levels, and rescuing the expression of reproductive genes. Studies show elevated PACAP levels in the brain prior to the critical proestrous phase inhibited ovulation [37]. In the case of intranasal application, PACAP alone prevented ovulation in about half of the rats [38]. An additional study demonstrated that PACAP stimulated the expression of the Kiss-1 gene, which codes for the neuropeptide kisspeptin, in two distinct hypothalamic cell types. Furthermore, the expression of PACAP in these cells was enhanced by the sex hormone estradiol. These findings suggest that PACAP may play a role in the feedback regulation of the female sex hormone estradiol [39]. Increased levels of circulating estradiol in PCOS may contribute to the overexpression of PACAP in hypothalamic cells. PACAP dysregulation and subsequent altered signaling of kisspeptin can cause abnormal secretion of GnRH pulse, which leads to increased LH/FSH ratio, thereby affecting androgen levels and ovulation.

A cross-sectional, matched-cohort study by Storch et al. found that women with migraine and regular menstrual cycles exhibited higher interictal PACAP-38 levels across the menstrual cycle compared to healthy controls. Conversely, women with migraine who were using combined oral contraceptives had PACAP-38 levels similar to those observed in healthy women [40]. This observation suggests that the concentration of circulating PACAP may be associated with the level of reproductive hormones, particularly luteinizing hormone which happens to be increased in women with PCOS. Additionally, among migraineurs, PACAP-38 concentrations were higher during physiological menstruation compared to the withdrawal bleeding associated with the hormone-free interval of COC use, suggesting that endogenous and exogenous sex hormones differentially impact PACAP-38 secretion [40].

Studies investigating PACAP in relation to migraines in women with PCOS have revealed important insights. Women with PCOS, who often experience hormonal imbalances, insulin resistance, and heightened inflammation, may have more circulating PACAP in their system and therefore, be particularly prone to PACAP-driven migraines [41]. Research shows elevated PACAP levels in migraine sufferers, and evidence suggests that PACAP exacerbates migraines through mechanisms like vascular dilation and neurogenic inflammation. Women with PCOS may have an intensified response to PACAP due to their metabolic and hormonal profiles, potentially leading to more frequent or severe migraines [42]. Based on these findings, several therapeutic approaches are being explored to target PACAP signaling pathways. PACAP antagonists, which block PAC1 receptors, can prevent PACAP from triggering the vascular and inflammatory processes associated with migraines. Monoclonal antibodies targeting PACAP or its receptors have shown promise in clinical trials, reducing migraine frequency and severity [43]. Additionally, small molecule inhibitors are being studied to block PACAP signaling at multiple levels, offering a potential personalized treatment for migraine in PCOS. These therapeutic strategies represent promising avenues for future migraine management in this vulnerable population [44].

Tryptophan/kynurenine pathway: its role in migraine and PCOS

Tryptophan is a vital amino acid that is crucial for neurological function, as it acts as the biochemical precursor to serotonin (5-HT). More than 90% of tryptophan in mammalian cells metabolizes in the kynurenine pathway rather than toward 5-HT [45]. A cross-sectional study by Wang et al. revealed that patients with PCOS exhibited elevated plasma levels of tryptophan and its metabolites, including serotonin, kynurenine, kynurenic acid, and quinolinic acid [2]. This abnormal activation is driven by increased activity of the rate-limiting enzymes IDO and tryptophan 2,3-dioxygenase (TDO) [2]. This suggests abnormal activation of the tryptophan catabolism pathway. Furthermore, the researchers determined that tryptophan metabolism in PCOS patients was skewed more toward the kynurenine pathway compared to the serotonin pathway [2]. Notably, this abnormal tryptophan metabolism was found to be associated with the pathophysiology of PCOS, even after accounting for the influence of obesity-related factors [2].

The researchers also observed that tryptophan, kynurenine, and quinolinic acid were significantly associated with C-reactive protein levels [2]. This can have an impact on the inflammatory state in PCOS patients. This inflammation may exacerbate insulin resistance and other metabolic disturbances. At the same time, kynurenine pathway metabolites can cross the blood-brain barrier, potentially influencing the hypothalamic-pituitary-gonadal (HPG) axis and contributing to hormonal imbalances [2]. Elevated metabolites were so closely related to the increased risk of PCOS that Wang et al. suggest a combination of tryptophan, kynurenine, and kynurenic acid could be used as potential markers to predict the risk of PCOS [2]. Furthermore, the altered tryptophan metabolism may have implications for mental health, as it could affect serotonin levels and increase the risk of mood disorders in women with PCOS. Yang et al.'s metabolomic analysis revealed that seven tryptophan metabolites were significantly elevated in patients with PCOS, indicating heightened activation of the tryptophan-kynurenine pathway in this condition [46]. Additionally, they found that most tryptophan-kynurenine metabolites were strongly associated with PCOS symptoms. In agreement with these results, an increase in tryptophan was also observed in follicular fluids and plasma from PCOS patients [47].

Recent research suggests that the kynurenine pathway and its downstream metabolites may contribute to migraine pathophysiology through multiple mechanisms. Metabolites like kynurenic acid and quinolinic acid have been implicated in inflammation in the nervous system. These compounds can affect immune cell function, particularly microglia, leading to the release of proinflammatory cytokines in the central nervous system [48]. Elevated levels of kynurenine metabolites are also linked to heightened oxidative stress and subsequent cellular damage, which are associated with migraine attacks. Additionally, certain metabolites from the kynurenine pathway can influence the activity of glutamate receptors, which are crucial in the development of migraines. This modulation of glutamate receptors by kynurenine metabolites may enhance neuroexcitability and contribute to the onset or exacerbation of migraine symptoms [49]. Furthermore, some kynurenine pathway metabolites can directly modulate the activity of glutamate receptors, which play a key role in migraine pathogenesis. This evidence underscores the potential involvement of the tryptophan-kynurenine pathway in migraine pathophysiology, further connecting this metabolic disturbance observed in PCOS to migraine susceptibility in this population.

Studies have reported that the prevalence of depressive symptoms among women with PCOS is substantial, ranging from 16% to 55% [50,51]. One study found that the kynurenine/tryptophan ratio, which is an indicator of serum IDO activity, was significantly higher in migraine patients with depressive symptoms [52]. It is possible that shared dysregulation of tryptophan metabolism contributes to comorbid migraine and depression in some PCOS patients. Recent research by Curto et al. has demonstrated an association between chronic migraine and dysregulation of kynurenine pathway metabolites [53]. They found a significant reduction in serum kynurenine and kynurenic acid levels in chronic migraine patients compared to controls. Tuka et al. observed a significant suppression in the entire tryptophan metabolic pathway during the interictal phase in individuals with migraines, while noting a trend of increased metabolite levels during the ictal phase [54]. Elevated concentrations of tryptophan, kynurenine, and kynureninic acid in the peripheral circulation may be a result of the migraine attack, potentially serving as a compensatory mechanism to reestablish energy homeostasis and shield the organism against deleterious effects.

Kynurenic acid, an endogenous NMDA receptor antagonist, along with its synthetic analog, has shown the capacity to inhibit PACAP overexpression, offering neuroprotective effects. The regulation of PACAP gene expression is primarily mediated by intracellular calcium homeostasis, which is controlled by NMDA receptors [55]. PACAP has been shown to strengthen the functional connection between neuronal nitric oxide synthase, considered a key factor in migraine development, and NMDA receptors in models of inflammatory and neuropathic pain [56]. Körtesi et al. discovered that blocking NMDA receptors can prevent PACAP overexpression in an experimental migraine model [57]. This finding supports the notion that treatments aimed at modulating glutamatergic transmission, such as kynurenic acid derivatives, could offer therapeutic benefits for migraine management.

The final metabolite of the 5-HT pathway of tryptophan metabolism is either MELA or 5-hydroxyindoleacetic acid (5-HIAA), and elevated 5-HIAA levels appear to be a contributing factor in triggering or exacerbating migraine headaches. Conversely, melatonin is a potent antioxidant that plays a role in the regulation of circadian rhythms. Nocturnal plasma samples from individuals with migraines revealed reduced MELA concentrations [58]. This reduction was linked to hormonal fluctuations [59]. Reduction in melatonin levels due to removal of the pineal gland and continuous exposure to light has been linked to the development of PCOS-like symptoms [60]. The significance of MELA in migraine became evident when patients experienced a decrease in headache frequency and intensity after receiving MELA; therefore, it is believed that migraine attacks may be a reaction to disruptions in pineal cycles [61,62]. Decreased melatonin concentration during the interictal period may trigger subsequent migraine attacks by removing its protective effects. After a migraine attack, peripheral melatonin levels may rise as the body attempts to restore normalcy and alleviate pain. These findings align with the observations made by Wang et al., who reported elevated plasma levels of tryptophan and its metabolites in individuals with PCOS. Jain et al. also found that patients with PCOS had significantly higher plasma melatonin levels that were positively associated with their serum testosterone levels, compared to age- and weight-matched controls [63]. This is significant given the higher prevalence of migraine experienced by PCOS patients compared to healthy individuals, suggesting a potential link between the dysregulation of the tryptophan pathway and the increased susceptibility to migraine attacks in this patient population.

## Conclusions

The identified involvement of PACAP and dysregulation of the tryptophan-kynurenine pathway in both migraine and PCOS provides insights into a potential mechanistic relationship between these two conditions. Specifically, alterations in PACAP signaling and the tryptophan metabolic pathway, including changes in melatonin levels, may contribute to the heightened migraine susceptibility seen in women with PCOS. Further research is needed to fully elucidate the complex interplay between these pathways and migraine pathogenesis in the context of PCOS.

The reviewed findings suggest that targeting metabolic pathways involving tryptophan and its derivatives, along with the potential use of kynurenic acid agonists may offer promising therapeutic approaches for managing migraine in PCOS patients. Additionally, PACAP-targeted therapies may hold promise for the treatment of both migraine and PCOS, though further research is warranted to explore this potential.
